# Adolescents’ Daily Activities and the Restorative Niches that Support Them

**DOI:** 10.3390/ijerph9093227

**Published:** 2012-09-05

**Authors:** Jenny J. Roe, Peter A. Aspinall

**Affiliations:** School of the Built Environment, Heriot-Watt University, Edinburgh EH14 4AS, Scotland, UK; Email: p.a.aspinall@hw.ac.uk

**Keywords:** adolescent, personal project, wellbeing, flourishing, restorative niche, place

## Abstract

This paper explores wellbeing from the perspective of the psychological dynamics underlying adolescents’ relationship with place. It uses a dynamic model of wellbeing called personal project analysis (PPA) which captures the concept of ‘flourishing’, defined as functioning well in your activities, strivings and interactions with the world [1]. Using PPA methods we identified adolescents’ daily activities and the ‘restorative niches’ that best support them. A series of settings (including home, urban and natural outdoor places) were explored using PPA with 45 young people (aged 11–13) living in Edinburgh, Central Scotland. Participants were asked to think of eight projects of current importance to them, to say where the project took place and to rate each project against a series of core wellbeing dimensions measuring project meaning, manageability, support and affect (how much fun, stress *etc*.). Latent class analysis was carried out to explore clusters—or sub-groups—in the data and to identify the significant discriminators between clusters. A three-cluster model produced the best fit with project type, project place and wellbeing indicators (fun and stress) significantly discriminating between the three clusters. The three clusters were labeled by their dominant environmental context, ‘faraway’ (e.g., beach, national parks, hills), ‘everyday’ (e.g., home, school, local streets) and ‘citywide’ (e.g., sport settings, urban town context). ‘Faraway’ and ‘citywide’ clusters had a significantly higher wellbeing content, especially for fun and stress; the ‘everyday’ cluster indicated local environs remain a dominant project place for this age group, but are associated with greater stress. We compare findings with adults and suggest that outdoor settings further afield from home have greater significance within adolescent project systems, but that support is needed to facilitate access to these places.

## 1. Introduction

### 1.1. A Social Ecological Framework for Measuring Human Flourishing

This study uses Brian Little’s model of person-environment relationships which explores wellbeing from the perspective of how well a person is flourishing in their personal projects. Personal projects are defined as “*extended sets of personally salient action in context*” [2] and can range from trivial pursuits (e.g., ‘tidying my room’) to more ambitious enterprises (e.g., ‘improving my mathematical skills’). Other related goal concepts include future goals [3], life tasks [4] and personal strivings [5] which are similar to—but not synonymous—with personal projects. Personal Project Analysis (PPA) [1] was developed to capture both the personal and environmental resources that impinge on project pursuit, and has been successfully used to explore older people’s relationships with the environment (particularly natural environments) and to identify what physical features support or hinder physical activity [6]. In this study, we use PPA as a framework to explore the world of adolescents aged 11–13 undergoing an important life transition—from junior to secondary school—and to identify the environmental niches that can help support their activities and wellbeing. 

**Figure 1:**
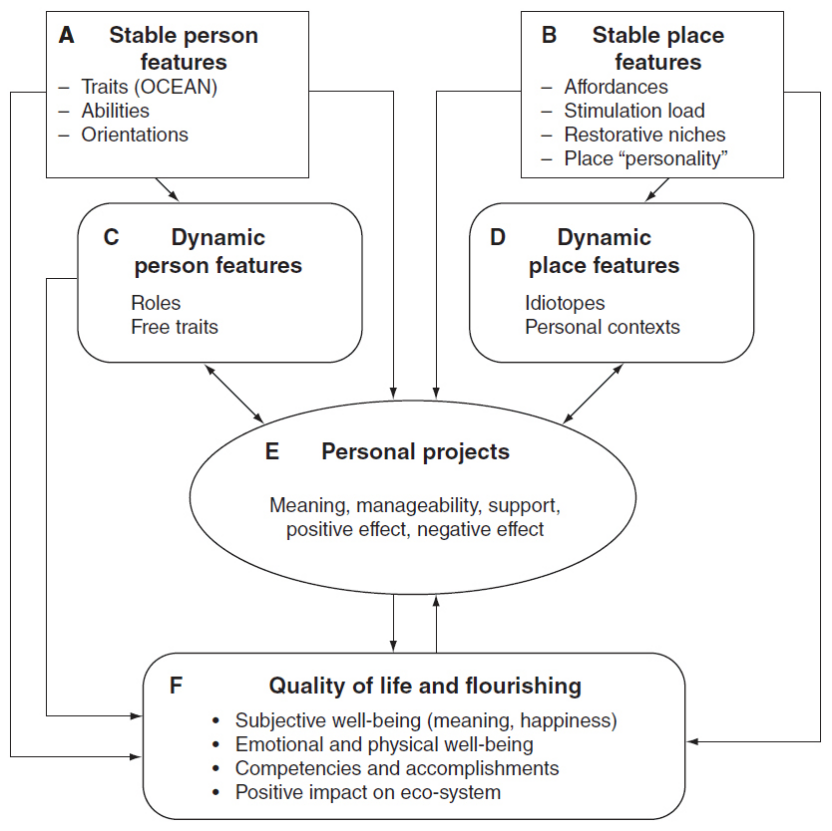
Social ecological framework for measuring human flourishing (reproduced, with permission from Brian Little [7]).

Little’s model posits that wellbeing is enhanced by the sustainable pursuit of core personal projects and that this requires both personal resources (e.g., self-esteem, resilience) and external resources (*i.e.*, the social ecological context) [7]. Figure 1 illustrates how person and place features (Boxes A, B, C and D) join together to impact on personal projects (Box E) and flourishing (Box F). Personal resources act on flourishing through ‘fixed traits’ (Box A) and ‘free traits’ (Box C). ‘Fixed traits’ refers to the five major factors underlying personality, captured in the acronym OCEAN (Openness, Conscientiousness, Extraversion, Agreeableness and Neuroticism); ‘free traits’ are those aspects of a person that enable them to act in a ‘non-typical’ way to achieve a particular goal (e.g., an introverted person acting in an extroverted fashion to advance a particular project). Secondly, external conditions act on flourishing via ‘fixed’ and ‘dynamic’ place features (Box B and Box D). Some ‘fixed’ features—the physical amenities of a place—are believed to support wellbeing. For example, natural environments that are high in soft ‘fascination’ (*i.e*., they hold just enough interest to promote reflection without demanding all of a person’s attention) are believed to promote psychological restoration [8]. Dynamic place features include the social context and the idiosyncratic adaptations an individual might make to a physical feature to achieve a particular goal (e.g., adolescents in the UK frequently adopt local bus shelters to support social gatherings). Finally, personal projects act as the conduit, through which person and place features impact upon flourishing (Box F). 

Flourishing is defined by the New Economics Foundation (NEF) as a state where people are “*functioning well in their interactions with the world and experiencing positive feelings as a result*” [9]. This definition of wellbeing reflects trends in positive psychology that wellbeing is more than simply life satisfaction or feeling happy; it also includes engagement (the feeling of being lost in a task or goal), relationships, meaning in life and accomplishments [10]. This broader definition of wellbeing is captured in PPA by measuring core project dimensions relating to project meaning, manageability, affect and support (Box E). In this way, it gives a richer picture of ‘how it’s going’ for an individual and reflects a more dynamic measure of wellbeing than simply asking about satisfaction with life. 

### 1.2. Restorative Niches

In this study we focus on the environmental context (*i.e*., the project place) impacting on flourishing, and particularly the concept of restorative niches, a ‘stable’ place feature that offers opportunities to promote wellbeing. Restorative environments (typically natural environments) have been shown to have important psychological health benefits that include raising mood and aiding recovery from fatigue and stress [11]. Although closely related, restorative niches differ from restorative environments in one important aspect [12]: whilst restorative environments are postulated as having universal benefits, a restorative niche takes on many forms and corresponds to a specific individual trait that needs restoration. So, for example, an extrovert might seek out a very different kind of restorative niche from an introvert. Korpela’s definition of restorative niches captures the need for a good fit between personal needs and a particular environment [13]. However, a person may act out of character so as to appear to fit a particular environment, taking advantage of that place only in order to pursue a particular project [12]. Whilst there are environments that appear to be universally restorative individual differences in personality means a ‘one niche fits all’ strategy cannot be applied. For the purposes of this paper, we are accepting Little’s model and conceptualising a ‘restorative niche’ as an environment that supports individual personal projects as measured by the core wellbeing dimensions of positive and negative affect, meaning, manageability and support. These five appraisal factors have consistently been shown to predict aspects of flourishing [7]. 

### 1.3. Young People’s Personal Project System

Being engaged in challenging, absorbing and meaningful projects has been defined as an integral component of wellbeing in young people [14] but there appears to be limited research linking personal projects to wellbeing in adolescents [15,16,17]. The most meaningful projects for adolescents (aged 13 to 18 years) are those relating to interpersonal relationships, whilst academic projects are the least meaningful [15]. The most manageable personal projects are those related to community action (societal projects) and sport; and the most stressful are those relating to academic and intrapersonal projects (or self-focused projects) [15]. Self-focused projects and future-education projects are related to greater stress in adolescents (aged 15); present-interpersonal projects (*i.e.*, leisure, friend, family and school related projects) are associated with higher levels of life satisfaction [16]. This reflects research in adults showing relationships between interpersonal projects and positive wellbeing [18] and between self-focused projects and stress and depression [19]. Where adolescent project systems appear to differ from adults is in the importance of the identity dimension (to what extent a project is pro-typical of someone). Identity projects have been identified as a central amongst the context of adolescent activities and goals [20]. Typically these are “*projects involving interpersonally intimate or nurturant themes*” [15] notably societal projects and family and friend relationship projects that are also linked with a greater sense of wellbeing. Identity projects are linked with greater feelings of efficacy and belonging in adolescents, and with higher rated subjective wellbeing [17]. 

## 2. Aim of Study

Whilst there are some studies of environmental drivers in adult project systems [21,22] to our knowledge there is no analysis of the environmental context impacting on adolescent personal projects (herewith referred to as ‘project places’). Owing to this gap, we did not form any a priori hypotheses but posited the following research questions:

(1) What sorts of personal projects are engaging adolescents living in urban Scotland?
(2) What does the project system tell us about the wellbeing of these adolescents?
(3) Are project places providing the restorative niche (an environment that supports individual personal projects) necessary to support adolescent wellbeing?

## 3. Method

### 3.1. Overview

Adolescents aged 11–13 (n = 45) were recruited from youth clubs in Edinburgh City, mostly located in deprived suburban fringes of the city, some 3–4 miles from the city centre. The Carstairs Scottish indices of deprivation [23] for these locations ranged from 4 to 6 indicating higher levels of deprivation than the average for Edinburgh City (3.42) (Carstairs is a widely used and well-validated indicator of area-level socio-economic deprivation in Scotland based on prevalence of household overcrowding, unemployment among men, low social class, and not having a car). Boys formed 58% of the sample and girls 42%; ethnicity was predominantly white Scottish (98%). Participants were self-selected to the study and signed consent from the adolescent and their parent/carer was a requirement to take part. The research was subject to approval by Heriot-Watt University’s School of Built Environment Research Ethics Committee. 

This was a cross sectional study design focusing on one phase of life. The adolescents in our sample had recently transferred from primary to secondary school and faced a particular set of age-related developmental tasks and challenges in adapting to a new school environment, increasing independence, and making the transition from childhood to adolescence. 

### 3.2. Project Elicitation

The data was collected during a 15-minute individual interview with each recruit. Interviews were structured around a simplified adaptation of the Personal Project Analysis Inventory [1] and were recorded, transcribed and coded as outlined below. Participants were asked to think of eight important projects replicating methods in earlier studies [22], rank them in order of importance and rate them against six project dimensions (see below). 

### 3.3. Measures

#### Project Measures

Six key project indicators were identified in discussion with Brian Little [24] and reflect the indictors in Box E, Figure 1. The first two indicators are affective dimensions and indicate how we feel about a project: (1) positive affect (*enjoyment*); (2) negative affect (*stress*). The remaining three indicators are cognitive appraisal dimensions indicating what we think about a project; (3) project manageability (levels of mastery and project *efficacy i.e*., the anticipated success of achieving the project goal); (4) project *support*; (5) project meaning* (*how *important) * and (6) *self-identity* (*i.e*., how expressive of you is this project) a dimension defined as particularly salient to young people’s well-being [15]. The young people were asked to rate each project on a scale of 1–4 ranging, for example, on the enjoyment scale from ‘*definitely fun*’ to ‘*definitely not fun*’. The environmental context was defined by asking participants to name the place where a project would take place. (We also asked ‘with whom’ a project would be carried out with but this data is not reported here). 

### 3.4. Scoring and Reliability

#### 3.4.1. Projects

The projects were classified into ten categories based upon content. The categories were similar to those used in previous studies on adolescents [15,16,17] with the addition of ‘new experiences’ particularly salient to this sample undergoing a major life transition from junior to secondary education. The ten project categories are detailed below with examples from participants italicized:

- Interpersonal-family (“*help my grandad, he’s quite old*”, “*get on better with my brother, be less annoyed with him*”)
- Interpersonal-friend (“*see more of my old friends*”, “*get on better with my mates’ girlfriend*”)
- Self-focused projects (“*getting more confident about meeting people*”, “*express myself, be a bit less shy*”)
- Societal (“*have a charity sale, maybe a street sale, I’ve done that before*”; “*take a group camping*”; “*help people less better off*”)
- Sport/Health (“*brush up my goal keeping skills*”, “*improve my fitness, get a wee bit more exercise*”)
- Education (“*get my homework done on time*”, “*get better at maths*”)
- Career (“*go to university, maybe physiotherapy*”, “*work in the theatre*”)
- Hobby (“*get better at cooking*” “*sing more*”)
- New Experiences (“*scuba diving*”, “*just explore and stuff*”, “*go to Barcelona*”)
- Autonomy (“*I would like to be able to walk or use public transport and use my bike more than ask for lifts*”)

#### 3.4.2. Places

Place categories were coded according to an earlier study [22] with some amendments to reflect settings particularly salient to young people. This generated seven categories of place: the home, school/youth clubs (the latter is often attached to the school in this context), city context (being in town), the local outdoors (streets, parks, playing fields, nearby woods), sport settings (formal), and faraway places (beach, hills, other cities). In this deprived urban sample, ‘faraway places’ represents all those places beyond the city centre (the nearest Scottish beach/mountain is just as inaccessible for these adolescents as further afield places like Barcelona even though in kilometres they are very much nearer). 

Classification reliability was checked on a proportion of the data, measured by the percentage rate of agreement between the two raters, with 93% agreement for projects and 96% for project places. Following methods in the literature [22,25], relative frequency scores were calculated separately per person for each place and project. For example, the place frequency was calculated by dividing the number of times a particular place category was mentioned by a subject by the total number of projects generated (8). The relative frequency score is generated from within the context of an individual’s overall project system, and offers a more appropriate basis for analysis, than the overall frequency calculated within the full sample. 

## 4. Results and Discussion

Firstly we report some descriptive statistics followed by latent class analysis (LCA) from Statistical Innovations [26] which was carried out to explore clusters—or sub-groups—in the data and to identify the significant discriminators between clusters. LCA is a novel way of classifying data to show person-environment relationships and has been used previously to explore adolescent physical activity and the built environment [27]. 

### 4.1. Descriptive Statistics

#### 4.1.1. What Sorts of Personal Projects are Engaging Young People Living in Urban Scotland?

The young people had no difficulty generating their projects or evaluating them on the wellbeing indicators. 77% of our participants were able to generate eight projects and the remainder seven projects. Table 1 below presents the relative frequency of project types as seen in the context of an individual project system. Sport/health projects for example, make up 32% of our participants project system. Interpersonal relationships with family, projects offering new experiences and self-focused projects were also frequently mentioned. We found statistically significant differences between boys and girls relating to project type (Mann Whitney U Test); boys project systems were more dominated by sport/health projects (*p* < 0.05); girl had a broader mix of projects with significantly more career, hobby and societal projects (*p* < 0.05). 

**Table 1:** Relative frequencies of project categories.

Project Type	Relative Mean Frequency (%) ( *SD*)		
	Total n = 45	Male n = 26	Female n = 19
Sport/health	0.32 *(0.17)*	0.35 (0.02) *	0.21 (0.09) *
Self focused	0.25 *(0.11)*	0.20 *(0.09*)	0.28 *(0.10)*
New experiences	0.24 *(0.09)*	0.25 *(0.09)*	0.21 *(0.07)*
Interpersonal (family)	0.23 *(0.09)*	0.24 *(0.09*)	0.21 *(0.09*)
Interpersonal (friends)	0.20 *(0.07)*	0.22 *(0.08)*	0.17 *(0.06)*
Societal	0.19 *(0.08)*	0.16 ( 0.06) *	0.23 (0.10) *
Career	0.18 ( *0.08)*	0.14 *(0.01*) *	0.23 *(0.10)* *
Hobby	0.17 *(0.07)*	0.13 (0.01) *	0.20 ( 0.08) *
Education	0.16 ( *0.05)*	0.17 *(0.05)*	0.13 *(0.01*)
Autonomy	0.14 *(0.08)*	0.13 *(0.01)*	0.15 *(0.02)*

* statistically significant gender difference, *p* < 0.05.

#### 4.1.2. How are Things Going for the Young People in Our Sample?

The dimensional project ratings provide an indication of how well that person is functioning and flourishing in their daily activities. The distribution of four wellbeing dimensions (*fun*, *efficacy*, *support*, *stress* and *self-identity*) was negatively skewed, *i.e*., the means lent towards higher values, indicating that wellbeing within the sample was above the median score (=2). Table 2 below shows the means for each dimension across each project type.

**Table 2:** Mean scores by project type.

Project Type	Fun Mean *(SD*)	Stress * Mean *(SD)*	Efficacy Mean *(SD)*	Identity Mean *(SD)*	Support Mean *(SD)*	Importance * Mean *(SD)*
Interpersonal family	3.22 *(1.10)*	2.39 *(1.13)*	3.25 *(0.94)*	2.84 *(1.31)*	2.75 *(1.30)*	6.07 *(1.79)*
Interpersonal friend	3.05 *(1.07)*	2.39 *(1.20)*	3.52 *(0.60)*	2.57 *(1.33)*	2.57 *(1.40)*	4.95 *(1.93)*
Self focused	2.74 *(1.20)*	2.74 *(0.99)*	3.19 *(0.98)*	3.17 *(0.97)*	2.88 *(1.20)*	4.57 *(2.06)*
Societal	3.53 *(0.81)*	2.20 *(1.16)*	3.34 *(0.94)*	2.75 *(1.22)*	2.74 *(1.21)*	4.69 *(2.07)*
Sport/health	3.84 *(0.52)*	1.87 *(0.94)*	3.32 *(1.19)*	2.87 *(1.19)*	2.79 *(1.33)*	3.90 *(2.15)*
Education	2.51 *(1.20)*	2.90 *(0.95)*	3.33 *(0.82)*	3.33 *(0.96)*	2.93 *(1.25)*	5.87 *(2.11)*
Career	3.65 *(0.67)*	2.55 *(1.05)*	3.57 *(0.59)*	2.72 *(1.22)*	3.55 *(0.74)*	5.03 *(2.39*)
Hobby	3.71 *(0.78)*	1.90 *(1.20)*	3.17 *(1.22)*	2.45 *(1.35)*	2.95 *(1.16)*	3.61 *(2.23)*
New experiences	3.69 *(0.78)*	2.25 *(1.01)*	3.05 *(3.05)*	3.10 *(1.13)*	2.48 *(1.32)*	3.51 *(2.36)*
Autonomy	2.60 *(1.14)*	2.25 ( *0.50)*	3.20 *(1.10)*	2.20 *(1.64)*	1.40 *(0.54)*	5.00 *(0.82)*

Note: a lower score on stress is positive; a higher score on identity means a project is less self-typical; a higher score all other project dimensions is positive.

The means provide an indication of what kinds of pursuits provide the greatest meaning, manageability and mastery for these young people. The most meaningful (important) activities are interpersonal and education projects, whilst the least meaningful are hobby and sports/health projects. The most manageable (best supported) projects are career projects whilst autonomy projects are very poorly supported. Easier to master projects (efficacy) are career projects; those most difficult to master are new experience-related projects. All projects were ranked as atypical projects (*i.e*., they were ranked above the median score of 2 and would cause ‘a bit’ or ‘a lot’ of surprise amongst family and friends), with self-focused and education projects rated as most untypical. The pursuits offering most fun are sport/health and hobby projects. Most projects were rated above average on the stress scale, with educational projects causing the greatest stress, followed by self-focused projects.

#### 4.1.3. What Types of Places are the Projects Being Carried Out in?

Our respondents mentioned on average five project places (with a range of two to eight), with the most frequently mentioned places being faraway places (mountains, the beach, overseas) and their own home (see Table 3 below). Fewer projects were placed in the town context (shops, cafes, museums) and recreational facilities. Although sports projects were amongst the most popular, they mostly took place in the natural outdoors (fields, local parks) rather than formal sports centres. We found statistically significant differences (Mann Whitney U Test) by gender, with boys more frequently mentioning the local neighbourhood (home, local outdoors) as project places (*p* < 0.05). 

**Table 3:** Relative frequencies of project places.

		Relative Mean Frequencies (%) ( *SD*)		
Project Places	Total n = 45		Male n = 26	Female n = 19
Faraway places (beaches, hills, mountains, overseas)		0.29 *(0.14)*	0.29 *(0.15)*	0.28 *(0.12)*
Home (friend, family)		0.29 *(0.19)*	0.32 (0.21) *	0.25 (0.15) *
Youth club/School		0.26 *(0.13)*	0.26 *(0.12)*	0.27 *(0.13)*
Local outdoors (street, fields, woods)		0.25 *(0.09)*	0.27 (0.09) *	0.21 (0.09) *
Sport centre’s (formal)		0.22 *(0.10)*	0.20 *(0.12)*	0.25 *(0.12)*
Town context (shops, cafes, museums)		0.19 *(0.06)*	0.17 *(0.06)*	0.25 *(0.21)*

* Statistically significant gender difference, *p* < 0.05.

### 4.2. Latent Class Analysis (LCA)

Following methods outlined by Aspinall [28] latent class analysis (LCA) was carried out using version 4.0 of Latent Gold [29]. This is a form of regression analysis which can handle non parametric data and which identifies clusters or sub-groups (latent classes) in a data set. In an exploratory analysis, such as this, with no precedents on which to anticipate the cluster number, we took the following approach: we estimated four latent class models, (from 1 to 4 clusters and entered the project indicators (stress, fun, efficacy and support, self-identity)), the project places and project types (as categorised in Section 2.4) and added the covariate of gender. In order to avoid converging on a local solution, a number of parameters were used to determine model fit. The main one was the Bayesian Information Criterion (BIC) which rewards parsimonious models [30]. This was then considered with respect to the first non-significant *p* value (following bootstrap); a high entropy value; few bivariate residuals over 2.0; and a 2 × log likelihood bootstrap difference (2LL) between two models for which there was no significant change. In this latter case of the 2LL test the model with fewer clusters was chosen as the final model.

#### 4.2.1. Results

A 3-cluster solution was chosen as the best fit to the data. This was based on an assessment across several criteria. The BIC values for the first one to four clusters were 1,664, 1,634, 1,647, and 1,669 respectively suggesting a two cluster solution as this corresponded to the lowest BIC value. However follow up bootstrap *p* values (based on 500 iterations) showed that the two cluster solution was marginally significant (*p* = 0.04) whereas the three cluster solution was marginally non-significant (*p* = 0.052) with the latter therefore a better fit to the data. Finally the test of model improvement of the three cluster over the two cluster solution was confirmed by the conditional bootstrap based on the log likelihood difference value (2LL) which has more general application for nested models [29]. This 2LL difference (25.97) between a three cluster and two cluster model was significant (*p* = 0.012) indicating that the three cluster model was a better fit than the two cluster model. The three cluster model also had high entropy (0.84), and only one bivariate residual above 2.0, whereas the two cluster model had three such values. This single high bivariate residual (2.7) was between stress and support showing the three cluster model did fail to adequately reflect this relationship. The three cluster model correctly classified 90% of cases; this compares with 92% for a two cluster model and of insufficient difference. Based on the above criteria, a 3-class model seemed the most appropriate. 

#### 4.2.2. Latent Class Profiles

In Table 4 below the significance of each variable is assessed in its capacity to discriminate between the three clusters. The R squared value indicates how much of a variable is explained by the cluster model. The *p* values show that project place, project type and the well-being indicators of fun and stress are all significant discriminators between the clusters. Efficacy and support are marginal indicators; self-identity and gender are not significant (and therefore not reported below). 

**Table 4:** Parameters discriminating between clusters.

	Cluster 1	Cluster 2	Cluster 3	Wald	*p* value	R²
**Project place**	−2.8602	1.2237	1.6365	25.8119	0.0040	0.2736
**Project type**	0.6685	2.1206	−2.7891	27.2128	0.018	0.3569
**Wellbeing Indicators**						
**Fun**	0.8350	−1.1092	0.2741	19.8820	4.8e–5	0.3581
**Stress**	−0.3752	0.6142	−0.2390	10.6165	0.0050	0.1273
**Efficacy**	−0.1408	−0.2838	0.4246	4.6883	0.096	0.0579
**Support**	−0.2797	0.0739	0.2058	3.3959	0.18	0.0304

The first row of Table 5 shows that the clusters are of approximately the same size *i.e*., 35% of participant cases are in cluster 1; 34% in cluster 2; and 30% in cluster 3. The table presents the mean item-response probabilities for each place and project characteristic together with the wellbeing measures. These indicate the probability of reporting a value above the median value within a class. 

**Table 5:** Latent Class Profile.

	Cluster 1	Cluster 2	Cluster 3
**Cluster Size**	0.3549	0.3442	0.3009
**Project Place**			
**1 home**	0.0038	0.3702	0.4583
**2 school/youth club**	0.0167	0.2188	0.0007
**3 town**	0.0647	0.0092	0.2578
**4 local outdoors**	0.0023	0.3080	0.0881
**5 sports settings**	0.1082	0.0008	0.1915
**6 faraway places**	0.8043	0.0931	0.0035
**Project Type**			
**1 interpersonal**	0.0576	0.6543	0.0206
**2 self-focused**	0.0001	0.0300	0.0148
**3 societal**	0.0007	0.0255	0.2900
**4 sport/health**	0.0013	0.0438	0.2930
**5 career/education**	0.0208	0.0001	0.0001
**6 hobby**	0.0006	0.0005	0.2202
**7 new experiences**	0.0001	0.0001	0.0490
**8 autonomy**	0.9189	0.2457	0.1123
**Wellbeing**			
**Fun**			
**1–3 (low-mid)**	0.0864	0.7272	0.1986
**4–4 (high)**	0.9136	0.2728	0.8014
**Mean**	3.8516	2.7511	3.6589
**Stress**			
**1–2 (low)**	0.6570	0.2593	0.6025
**3–4 (high)**	0.3430	0.7407	0.3975
**Mean**	2.0408	2.8382	2.1500
**Efficacy**			
**1–3 (low-mid)**	0.4985	0.5596	0.2735
**4–4 (high)**	0.5015	0.4404	0.7265
**Mean**	3.2441	3.1514	3.5852
**Support**			
**1–2 (low)**	0.5727	0.4221	0.3680
**3–4 (high)**	0.4273	0.5779	0.6320
**Mean**	2.3218	2.6912	2.8238
**Covariates: gender**			
**Female**	0.3608	0.4143	0.4545
**Male**	0.6392	0.5857	0.5455

Table 5 shows that, if you’re in Cluster 1—reading down the column—you have a good chance of experiencing positive wellbeing; a high probability that your projects are fun (91%), low in stress (66%) but there is less chance of mastering them (49% efficacy rating) and receiving support (42% support rating). If you’re in Cluster 1 there’s a strong probability you’re male (64%), a 92% chance that your projects are autonomy related, and an 80% chance that they take place in faraway places. This cluster is therefore very much dominated by one project type and one project place. We labeled this cluster by its dominant environment *i.e*., ‘faraway’.

Cluster 2 indicates a good chance of having lower wellbeing; a higher chance your projects are not fun (72%) and stressful (74%) but that they are better supported (58%) with a reasonable chance of project mastery (56% efficacy). There’s a high probability your projects are interpersonal (65%) and are situated in the local environment (home, school, local outdoors) (90%). You have slightly higher chance of being male (59%) in this cluster. We have labeled this cluster by its dominant setting, ‘everyday’. 

**Figure 2:**
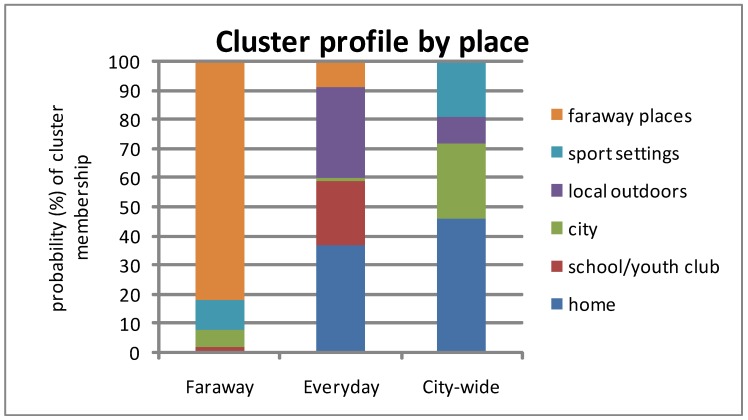
Cluster membership based on project place.

**Figure 3:**
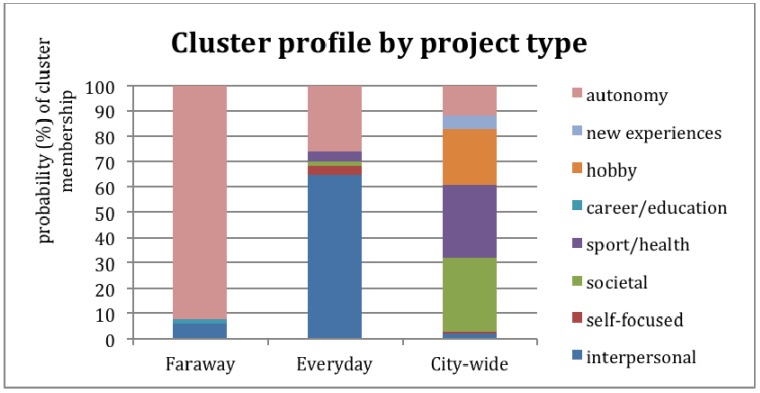
Cluster membership based on project type.

**Figure 4:**
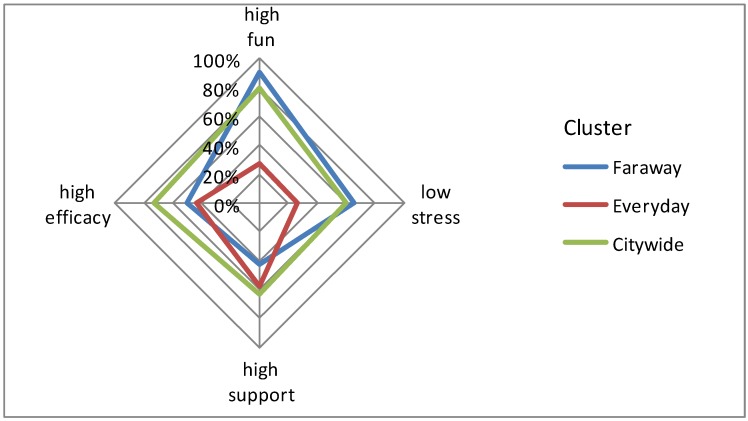
The probability of experiencing high wellbeing by cluster membership.

If you’re in Cluster 3, there’s a good chance you have higher wellbeing; there’s an 80% chance that your projects are high in fun (80%), low in stress (60%), and that you can master them well (73% efficacy) and are well supported (63%). Your projects are more likely to be spread across several categories (hobby/sport/health/societal) and take place in wider range of city-wide settings (sports centre and the city centre (cafes, shops, restaurants, cinema). We have labeled this cluster, ‘city-wide’ although the home is still a distinct project place within it. The spread of projects and places by cluster type is shown in Figure 2 and Figure 3, with the probability of experiencing ‘high’ wellbeing (*i.e*., above the median score for each dimension) illustrated in Figure 4.

Figure 4 shows higher wellbeing is associated with the cluster labeled ‘city-wide’; positive wellbeing (more fun/less stress) is also associated with the cluster ‘faraway’ but there is less chance of experiencing mastery and support. The cluster ‘everyday’ has the lowest wellbeing profile across all dimensions with the exception of support. 

### 4.3. Discussion

This paper asked, firstly, what sorts of projects are young people living in urban Scotland engaged in? Results show adolescents aged 11–13 focus on sport/health, interpersonal (family and friend), self-focused projects, new experiences and education-related goals, partially reflecting earlier research [15]. A new finding is the emergence of autonomy-related projects reflecting a growing need for independence in this particular age group. Gender differences partially reflect earlier findings [16] with girls reporting more self-focused, societal, and career related projects and boys more sport/health and education projects. Future career and acquisition projects did not emerge as major project categories, a significant difference with the older teens (aged 15+) [16] and adults [19]. Another difference with the older adolescent age group is that interpersonal goals focus on family and not friends; the context of family and home is therefore still very significant in this younger adolescent age group. When comparing results with studies in adults [19], societal and autonomy-related projects form a much greater part of young people’s project systems. Adolescents aged 11–13 appear to be more adventurous and altruistic in their goals as compared with adults, un-materialistic in their aspirations and not yet thinking about future careers. 

Secondly, we asked what can the personal project system tell us about the wellbeing of adolescents? The above average scores for wellbeing suggests that the adolescents in our sample are flourishing reasonably well; they appear to be enjoying their projects, feel well supported, able to do them well, and are developing new identities through their projects (friends and adults would be very surprised to see them do such things). Whilst this is a small sample, these findings support wider research in Scotland showing good levels of emotional wellbeing in young people [31]. Cluster analysis showed a mixed project system—carried out across a series of settings—is associated with higher overall wellbeing (‘city-wide’ cluster). Societal projects make a significant contribution to this cluster and are linked in the literature with higher wellbeing in adolescents [15]. The young people in our study have wide-ranging altruistic goals (e.g., ‘*have a charity sale*’, ‘*help my granddad*’, ‘*teach people how to ride a bike*’) and offer a rich source of potential social capital if the right support can be provided. The ‘faraway’ cluster—dominated by one project type (*i.e*., autonomy related projects)—ranked highly on affective wellbeing (high fun, low stress) but lower for levels of support. Surprisingly, interpersonal projects—dominating the ‘everyday’ cluster—and associated in the literature with higher wellbeing in adults—were not highly ranked on the wellbeing valuation measures with the exception of support. This project category is dominated by family projects—particularly the need to improve sibling relationships—a project pursuit which may be extrinsically driven (e.g., by parental demands) rather than intrinsically motivated, a dimension associated with higher wellbeing. This cluster also includes self-focused projects reflecting results in adults showing a relationship between this project type and stress [19]. 

Of interest, is the finding that self-identity (as defined by “typical self”) did not emerge as a significant discriminator between clusters—although this does not necessarily mean it is unimportant in adolescent project systems. Self-identity may be a non-significant discriminator of clusters because it is equally important across all clusters (for instance, in LCA typically ‘price’ in purchasing behaviour does not appear to discriminate between clusters since it is equally important). The projects generated by our sample were largely ranked as “un-typical” (*i.e*., projects likely to surprise parents and friends) and were spread across all three clusters. Those ranked most “untypical” were education-related and self-focused projects and were associated with higher stress valuations (Table 2). This confers with Little’s hypotheses [15] that a predominance of untypical project in adolescence may be linked with alienation and depression; however, acting in a “non-typical” way might also be construed as beneficial to adolescent wellbeing in that it allows young people to forge new identities. The inclusion of a depression scale in subsequent research would help establish these relationships more firmly. 

Thirdly, we asked are project places providing the restorative niches necessary to support adolescent wellbeing? The two clusters characterized by further afield places (‘faraway’ and ‘cityside’ settings) offer restorative niches that support project efficacy and positive and negative affect. The opposite setting—the home and local environs—is associated with higher levels of project support but ranked lower on emotional scales (*i.e*., fun and stress). There appears to be a tension in this age group between aspirations to explore and roam and the reality of having to stay local owing to difficulties in accessing places further afield (not having a car is one of the deprivation indicators for this sample, see Section 3.1). The literature points to the everyday context (*i.e.*, home, workplace and sport settings) as the heart of the adult project system [22]; the emergence of further afield places as an environmental context for adolescent projects is a new finding. This suggests an age-specific developmental need in young people for exploration not evident in the goal research in adults. Exploration, risk and adventure are known to be precursors of identity achievement in young people [32] and of adolescent wellbeing [14]. But without support (and specifically transportation) to facilitate access away from home environs (e.g., by the continuing support of youth club provision) this need for exploration and adventure will not be met in adolescents from deprived urban communities. 

The New Economics Foundation has flagged the need for developing appropriate tools for measuring wellbeing and flourishing in context and to understand the environmental drivers of wellbeing [9]. We suggest PPA is an ideal tool for this analysis, since it conjoins assessments of personal resources with the social-ecological context. PPA also taps into models of wellbeing currently being developed in the UK that build on self-determination theory [9], reflecting the three basic psychological needs of autonomy, relatedness and competence [33]. Competence (a sense of efficacy and believing you are making a meaningful impact on the world) is captured in the management dimensions of PPA (e.g., importance and efficacy); relatedness (a feeling of being connected to those around you) is captured in the support measure and ‘with whom’ dimension, and autonomy (feeling in control of your actions) is reflected in the support dimension (e.g., being able to get along with something on your own). PPA also offers a flexible method that can be tailored to capture age-appropriate wellbeing dimensions, for example, the identity dimension in adolescence was easily integrated into this study; in studies in older people, more appropriate age-related measures of wellbeing can be incorporated. An additional benefit of the method is participant engagement in the research process; the young people in our study happily engaged in project elicitation and were often surprised and delighted by the outcomes. 

#### Limitations

There are several limitations of the research. Firstly, the study was cross-sectional. A longitudinal approach would allow the relationship between personal projects and environmental resources to be mapped over time, and flag changes in the project system over adolescent transition periods. Secondly, our sample size was small, and there is an obvious need to replicate the findings in a larger sample. Thirdly, we were unable to show how traits impact on personal projects and restorative niches. In future research we recommend the inclusion of personality trait scales (e.g., OCEAN) in order to establish how personality discriminates between cluster profiles. The concept of restorative niches could be strengthened by having participants rate their project places for key restorative attributes, using the Perceived Restorativeness Scale (PRS) [34]. Finally, we were unable to show any direct link between supportiveness of the environment and wellbeing; this might be addressed in future research by asking to what extent a project place facilitates or hinders project pursuit [19]. 

## 5. Conclusions

This study has captured the rich complexities of adolescents’ daily activities as they make the transition from junior to secondary school. To our knowledge, this is first time PPA has captured wellbeing and its environmental context in the 11–13 age group. We have also shown the novel application of latent class analysis in exploring person-environment relationships. Further afield places (‘faraway’ and ‘citywide’ settings) are restorative niches that support project management and emotional wellbeing; however, everyday settings (home, school, local outdoors) remain a dominant environment, offering higher levels of project support but greater levels of stress. Whilst the projects elicited cannot be a true representation of all adolescent pursuits and passions, what emerged from our study is a rich ‘initial probe’ into the adolescent project system and the links between project places and wellbeing. 
